# In Vitro Evaluation of Bioavailability of Cr from Daily Food Rations and Dietary Supplements from the Polish Market

**DOI:** 10.3390/nu16071022

**Published:** 2024-03-31

**Authors:** Piotr Bawiec, Jan Sawicki, Paulina Łasińska-Pracuta, Marcin Czop, Ireneusz Sowa, Paweł Helon, Karolina Pietrzak, Wojciech Koch

**Affiliations:** 1 Department of Food and Nutrition, Medical University of Lublin, 4a Chodźki Str., 20-093 Lublin, Poland; piotr.bawiec@wp.pl (P.B.); paulina_lasinska@interia.pl (P.Ł.-P.); karolinapietrzak94@gmail.com (K.P.); 2 Department of Analytical Chemistry, Medical University of Lublin, 4a Chodźki Str., 20-093 Lublin, Poland; jan.sawicki@umlub.pl (J.S.); ireneusz.sowa@umlub.pl (I.S.); 3 Department of Clinical Genetics, Medical University of Lublin, Radziwiłłowska 11 Str., 20-080 Lublin, Poland; marcin.czop@umlub.pl; 4 Branch in Sandomierz, Jan Kochanowski University of Kielce, Schinzla 13a Str., 27-600 Sandomierz, Poland; phelon@ujk.edu.pl

**Keywords:** trace elements, diets, bioavailability, ICP-OES, GF-AAS

## Abstract

Only some of the nutrients consumed with food are able to be absorbed from the gastrointestinal (GI) tract and enter the systemic circulation (blood). Because some elements are essential minerals for humans, their beneficial effect on the body depends significantly on their bioavailable amount (the fraction that can be absorbed and used by the organism). The term bioavailability, which is very often used to describe the part of nutrients that is able to be absorbed, is influenced by various factors of exogenous and endogenous origin. The main purpose of the study was to assess the relative bioavailability of Cr from selected dietary supplements in the presence of various types of diets, which significantly influence the level of bioavailability. The research was performed using a previously developed and optimized two-stage in vitro digestion model using cellulose dialysis tubes of food rations with the addition of pharmaceutical products. Cr was determined using the ICP-OES and GF-AAS methods, depending on its concentration in particular fractions. The determined relative bioavailability ranged between 2.97 and 3.70%. The results of the study revealed that the type of diet, the chemical form of the molecule, and the pharmaceutical form of preparations have a significant influence on the bioavailability of Cr.

## 1. Introduction

Food of plant and animal origin is a major source of various nutrients, including trace elements, which are essential to humans and, therefore, must be consequently delivered to the body [1,2]. The only significant source of these compounds is food, which is mostly of natural origin. Recently, the role of dietary supplements as an important source of trace elements has rapidly increased; therefore, they should be considered an important complement to our daily, natural diet, which is crucial for medical, nutritional, and socio-economic reasons [3,4]. The importance of particular trace elements to human health is related not only to the amount consumed but also to the portion that can be absorbed and then used or stored in the body [5,6]. For food samples, oral bioaccessibility is very often estimated—it describes the maximum amount of a substance that was theoretically released in the gastrointestinal (GI) tract from the food matrix (bioaccessible fraction) and is ready to be absorbed [7]. Therefore, sometimes it is misleading, as both bioaccessibility and bioavailability terms are used for similar experiments [5,8,9,10].

Bioavailability and bioaccessibility are influenced by the complex food matrix and the chemical forms of particular molecules. However, the physiological condition of the GI tract and other parameters, like age, gender, or state of health, also significantly affect the level of bioavailability, which can be assessed only in models using human studies. However, considering ethical issues, especially when bioavailability is evaluated for toxic elements (Hg, Pb, or Cd) that are harmful to an animal or human body, such practices can be evaluated as unethical [5,11], and thus, in vitro models are considered to be more appropriate. In vitro techniques using simulated gastrointestinal digestion are easy to perform, cheap, and free of ethical concerns. Most importantly, such studies accurately imitate the conditions in the digestive tract, such as temperature, agitation, pH, and enzyme composition. Thus, these techniques are widely used for preliminary evaluation of the value of the bioavailability of elements from food, including dietary supplements [5]. There are multiple variations of these methods, including measuring bioaccessible fractions [5], studies using human GI microbiota cells [12,13], studies using semi-permeable cellulose membranes with specific pore sizes, imitating human intestines [8,9,14], and experiments using human Caco-2 cells [15]. All these methods have certain advantages and disadvantages. However, the lack of ethical issues, relative simplicity, and wide range of various modifications make in vitro studies widely used in the assessment of trace element bioavailability from different pharmaceutical and food products, including dietary supplements [8,9,16]. It is obvious that in vitro methods, which are based on chemical or biological experiments conducted outside the human body, do not ideally reflect the conditions in the GI tract, but by selecting the appropriate conditions for the enzymes’ work, temperature, and time, we are able to reproduce the conditions in the digestive tract with a high degree of probability. Therefore, according to numerous authors, simulated in vitro digestion methods using cellulose dialysis membranes are currently the gold standard for assessing the bioavailability of trace elements from food and pharmaceutical preparations [8,9,17].

Although Cr is still considered a trace element essential to the human body, its role has recently been strongly questioned. According to Vincent [18], there is not enough evidence that the absence of Cr in the diet might cause any deficiency symptoms. Moreover, there is no reliable proof that GTF (Glucose Tolerance Factor), the main enzyme associated with the positive effect of chromium on carbohydrate metabolism, exists [19]. Therefore, in 2014, EFSA issued a recommendation that no reference intake standards should be established for this element [18]. The current Polish population nutrition standards do not regulate the intake of this element [20]. In the USA, the recommended intake was established at 25 µg/day and in France at 60 µg/day [21]. Moreover, it was suggested that chromium, which is present in the human diet mainly from food processing (e.g., the use of stainless steel), was probably present in the diet of our ancestors in much smaller amounts than it is at present, so the current consumption levels of this metal should be regarded as unnaturally high. Therefore, it is difficult to talk about the symptoms associated with a deficient intake of chromium in our diet [18,22]. Although its biological role and essentiality to humans are unclear, the position of Cr in the dietary supplement market is very strong. It is still one of the most popular trace elements consumed in the form of various pharmaceutical products, especially by people who are overweight, obese, or have dyslipidemia or type-2 diabetes issues [23,24,25,26]. Therefore, although Cr’s position as an essential nutrient was recently questioned, its strong position in the food and pharmaceutical markets creates a need to evaluate its bioavailability in various food products.

Most studies on the bioavailability of Cr evaluate the influence of chemical form, dose, and differences between various food products. However, in the scientific literature, there are no data on the influence of whole, complex, and reconstructed diets on the absorption of Cr from dietary supplements. Also, the influence of the form of the pharmaceutical preparation (tablets and capsules) on the bioavailability of Cr has not been evaluated. Therefore, the main aim of the present study was to evaluate the relative bioavailability of Cr from dietary supplements in the presence of reproduced diets. For this reason, and considering that a well-balanced diet should contain foods from all major groups, reconstructed daily food rations (DFRs) were used in the study. These daily diets were used in the form of homogenates consisting of four meals and reflected typical types of human nutrition. The creation of such a research model enabled the analytical assessment of the impact of diet on the bioavailability of Cr from dietary supplements. To the best of our knowledge, this is the first study in which the bioavailability of Cr was assessed under the influence of complex model diets using a two-phase simulated digestion procedure, cellulose membranes, and analytical determinations by means of ICP-OES and GF-AAS methods. The latter analytical techniques were used as the most appropriate, considering their low detection limits and minimization of interferences. Moreover, the level of bioavailability of Cr from the studied dietary supplements was also evaluated, considering Cr chemical forms and pharmaceutical forms of the studied products in the presence of various types of diet.

## 2. Materials and Methods

### 2.1. Chemicals and Reagents

Nitric acid, hydrochloric acid, and hydrogen peroxide, which were used during the digestion stage and spectrometric determinations, were of Suprapur Grade and were bought from Merck (Darmstadt, Germany). The standard solution of Cr (1 mg/L) was bought from PlasmaCAL (SCP SCIENCE, Baie-D’Urfe, QC, Canada). Pepsin, pancreatin, and dialysis tubes, which were used to perform the simulated digestion stage, were purchased from Sigma-Aldrich (St. Louis, MO, USA). Sodium bicarbonate was bought from Avantor Performance Materials (POCH, Gliwice, Poland). During all determinations, high-purity deionized water (resistivity of 18.2 MWcm) obtained using an Ultrapure Millipore Direct-Q-R 3UV (Millipore, Bedford, MA, USA) was used. During the digestion stage, the DigiTubes and DigiFilters (SCP SCIENCE, Canada), certified for trace element analysis, were used.

### 2.2. Materials

#### 2.2.1. Dietary Supplements

Six dietary supplements containing Cr were used in the study. Particular products were chosen to represent the most prevalent chemical forms of Cr. The popularity of these dietary supplements among Polish consumers was also an important factor to take into account when selecting individual products. The inclusion criteria were as follows: dietary supplements in the “DS” category of the products, wide availability on the pharmaceutical market, and products within the expiration date. The following exclusion criteria were considered: difficult accessibility during research and the OTC (over-the-counter) category of the products. The Polish market for dietary supplements containing Cr is very broad; therefore, the study covered only products from reputable producers with an established position in the market. The aim of the study was not to evaluate the quality of individual products and their producers; thus, trade names of particular products were not provided, and each dietary supplement used in the current study was only marked with a specific number. The detailed characteristics of the supplements used in the research are presented in Table 1.

For each product, three subsamples were taken from three different series. All determinations were made in triplicate. This study was conducted in 2021–2022. The bioavailability of Cr was evaluated in 21 different experimental models. Considering the number of series, subsamples from each series, and repetitions, in total, 171 analytical samples were determined.

#### 2.2.2. Reconstructed Diet Duplicates

All food products consumed daily in terms of their quality and quantity are collectively called a diet, which is an appropriate form of human nutrition based on the basic principles of dietetics. Each diet has a specific nutritional value, i.e., the content of energy and individual nutrients. The diet is a major source of nutrients that are essential for the proper functioning of the body. A complex matrix of various food products taken during the consumption of meals influences the absorption of particular nutrients, both in a positive and negative way [27]. Thus, to estimate the bioavailability of Cr in the presence of different types of diets, three types of diets that are the most frequently used in the nutrition of healthy people were developed based on dietary scientific literature data and professional dieticians’ experience [27,28,29]. They were reconstructed and used in the experimental part of the present study. Three types of human diets were developed and reconstructed—standard, basic, and high-residue diets. The nutritional value of each diet was calculated using Dieta 6.0 software [National Food and Nutrition Institute, Warsaw, Poland]. The detailed characteristics of the DFRs used in the study, regarding their composition and nutritional value, are presented in the Supplementary Material (Tables S1 and S2).

The exact procedure that was developed to prepare and reconstruct DFRs was previously described in detail [30]. Briefly, all food products came from the retail market in the Lublin region. The majority of them (cereal products, milk and milk products, meat and meat products, eggs, vegetables, and fruits) were of local origin. The dishes were prepared and cooked in the laboratory according to local culinary practices, based on the guidelines presented in the available literature, and using a procedure that had been previously checked and validated [30,31,32,33,34]. The qualitative and quantitative composition of all DFRs used in the study was identical to those designed, reflecting the actual diet of the population. To reduce the risk of cross-contamination, laboratory equipment was thoroughly cleaned each time beforehand using lab-grade detergent, hot tap water, a low-concentration acid solution of Suprapur Grade, and ultra-pure deionized water. Reconstructed DFRs were weighed and homogenized in a homogenizer (Zelmer, Poland) with titanium blades and stored at −20 °C prior to analysis.

### 2.3. Two-Phase Enzymatic Model of In Vitro Digestion

In vitro models simulating the human digestive system are used to study the relative bioavailability of nutrients and drugs. Studies of this type under normal conditions are significantly hindered by problematic access to the lumen of the gastrointestinal tract, especially in the small intestine. To facilitate the study of the relative bioavailability of nutrients, models consisting of a two- or three-stage digestive system were created: the stomach small intestine, the oral cavity stomach small intestine, and the stomach small intestine large intestine. In the current study, one of the first digestion models proposed and optimized by Miller et al. [35] was used. It is a two-phase gastro-small intestine digestion model that uses appropriate digestive enzymes at the appropriate temperature and pH of the system in which the enzymes can be active. The original method was significantly improved, especially through the introduction of cellulose dialysis tubes, which are characterized by a special cut-off point that allows these membranes to be impermeable to high-molecular-weight compounds and thus better simulate the natural conditions in the gastrointestinal tract [14]. The procedure used in the present study was improved and previously checked and validated for its accuracy in evaluating the bioavailability of various food components, including trace elements. All the details were described elsewhere [30,36].

**Figure 1:**
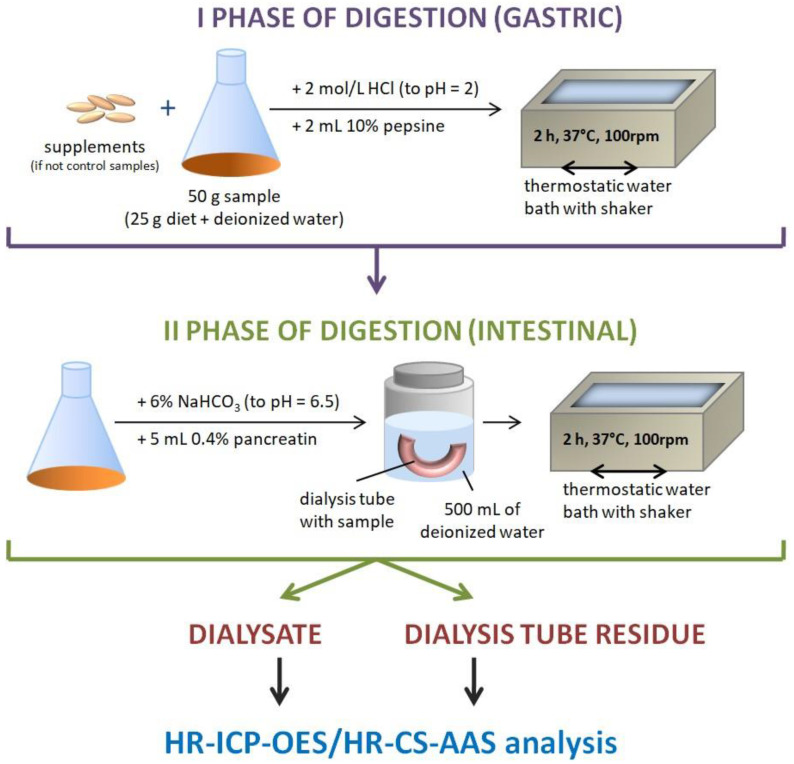
The scheme of the digestion procedure.

Briefly, in the present method, 25 g of the sample (homogenized diet) was weighed and filled with deionized water to 50 g. Such a model, in which there was no dietary supplement added, was used to evaluate the bioavailability of Cr from each model diet used in the study (standard, basic, and high-residue diets). In all models where dietary supplements were added, 25 g of the diet sample was weighed and mixed with one pharmaceutical portion of each product (tablet or capsule). Later, the whole system was filled with deionized water to a mass of 50 g and subjected to gastrointestinal digestion, which involved gastric digestion using pepsin in an acidic environment for 2 h in a thermostatic water bath with a shaker (Vibra, AJL Electronic, Kraków, Poland) at 37 °C. The samples were later transferred to cellulose dialysis tubes with a molecular weight cut-off (MWCO) of 2 kDa, made alkaline from pH 2 to 6.5 using 6% NaHCO_3_ solution, and further digested using pancreatin solution in 0.1 mol/dm^3^ NaHCO_3_ by shaking for 2 h in a thermostatic water bath at a temperature of 37 °C. This step, which imitated intestinal digestion, was performed in a polypropylene container with 500 mL of deionized water. The completion of the entire procedure allowed the attainment of two fractions: the dialysate—the solution surrounding the cellulose dialysis tube and the residue in the cellulose dialysis tube. Control samples were analyzed simultaneously using the same procedure. The obtained fractions were subjected to further analysis. Schematic illustration of the experimental procedure was presented in Figure 1. 

### 2.4. Analytical Determination of Cr

The obtained samples (both fractions—the dialysate and the tube reside) were digested as previously described [30]. Briefly, DigiPREP MS (SCP Science, Canada) equipment with a condensate recirculation system was used. The dialysate solutions (5 mL) were digested using 1 mL of concentrated (65%) HNO_3_ for 120 min at the temperature of 120 °C, whereas a mixture of 65% HNO_3_ and 30% H_2_O_2_ for 120 min at the temperature of 120 °C was used to perform digestion of the cellulose dialysis tube residues. The obtained and cooled digest solutions were filtered using a Rocker 300 vacuum pump (Rocker Scientific, New Taipei City, Taiwan) and DigiFILTER filters and then made up with deionized water to a final volume of 10 mL. The concentration of Cr was determined using the ICP-OES method with a PlasmaQuant 9000 Elite, a high-resolution inductively coupled plasma optical emission spectrometer (Analityk Jena, Jena, Germany). Moreover, a high-resolution atomic absorption spectrometer with a continuous light source (HR-CS-AAS), ContrAA 700 (Analytik Jena, Germany), and atomization in a graphite furnace (GF-AAS) was used for the analysis of samples with a low chromium content (below LOQ in the ICP-OES method). The determinations were performed in triplicate. Instrumental settings were presented in Tables S3 and S4 in the Supplementary Material. The equipment was calibrated using PlasmaCAL (SCP SCIENCE, Canada) Cr standard solutions (1000 µg/mL) with appropriate dilutions.

The applied analytical protocol has already been validated and checked for accuracy and precision in the determination of trace elements, including Cr [33,37]. However, because a different method of digestion of organic material was used, it was re-checked for its suitability for the determination of Cr in the investigated samples. For this reason, a mixture of flour and milk powder (7:3 *w*/*w*), fortified with specific and known amounts of various elements, including Cr, was used. The studied samples and the reference material were investigated in parallel under the same conditions. The analysis of the reference material was performed in six repetitions, and the obtained results are shown in Table 2.

### 2.5. Calculation of the Relative Bioavailability Value

Based on the obtained analytical data regarding Cr concentrations in dialysate solutions and cellulose tube residues obtained after the digestion process, the values of relative bioavailability (expressed as percentages) were calculated using the following formula:

$$B\%=\left(\frac{D+D_{r}}{T+D}\right)\times 100\%$$

where B% represents the bioavailability of Cr (%), D and T represent the amount of Cr (mg) in the dialysate and in the digest of the dialysis tube residue, respectively, whereas D_r_ represents the concentration of Cr (mg), corresponding to the equilibrium of concentrations on both sides of the cellulose membrane present inside the dialysis tube.

D_r_ was elaborated based on the following formula:

$$D_{r}=\frac{\left(C_{d}-C_{k}\right)\times V_{t}\times R}{1000}$$

where C_d_ is the concentration of Cr in the dialysate solution (μg/mL), C_k_ is the concentration of Cr in the control sample (μg/mL), V_t_ is the volume of the dialysis tube (mL), and R is the dilution factor.

### 2.6. Statistical Analysis

All data obtained in the study were statistically elaborated using Statistica v. 13.0 software (StatSoft, Kraków, Poland) and MS Excel 2010 (Microsoft). In order to present the obtained results, descriptive statistics methods, i.e., arithmetic mean (x, M), median (Me), standard deviation (SD), quartile range (IQR), minimum (Min), maximum (Max), and (F) and (H) test statistic values for the ANOVA test, were used to present the obtained data on a quantitative scale. To assess the compliance of the distribution of the studied variables with the normal distribution, the Shapiro–Wilk test was used. Parametric tests were used for data with a normal distribution of variables, and non-parametric tests were applied in the absence of a normal distribution.

The analysis of variance—one-way ANOVA—with Tukey’s post hoc test was used to assess statistically significant differences between several groups in the case of analyzing data on the relative bioavailability of Cr from dietary supplements. For multiple group data, when the influence of chemical forms of Cr in dietary supplements as well as pharmaceutical forms of the preparations used on the value of relative bioavailability was analyzed, one-way ANOVA with Tukey’s post hoc test and Kruskal–Wallis rank with Dunn’s post hoc test were applied. As the critical significance level (α) for all tests, a value of 0.05 was assumed (α = 0.05). In the current study, the following levels were set: *p* < 0.05—statistical significance; *p* < 0.01—strong statistical significance; *p* < 0.001—very strong statistical significance.

## 3. Results

### 3.1. Bioavailability of Cr under the Influence of Various Diets

The main objective of the present study was to estimate the impact of various types of diets (representing standard, basic, and high-residue diets) on the bioavailability of Cr from selected dietary supplements using a two-phase in vitro digestion model and analytical determinations of the element in the obtained fractions. Additionally, the assessed parameters also took into account the influence of the chemical formula and pharmaceutical form on the value of relative bioavailability. Table 3 presents the results of Cr bioavailability from investigated dietary supplements under the influence of various types of diets used in the study. The data obtained in the model in which no dietary supplements were used represent the bioavailability of Cr from a specified diet. For these samples, relative bioavailability was within the range of 2.97–3.70%. The highest value was obtained for the basic diet (3.70%) and the lowest for the standard diet (2.97%). The results for the standard diet were significantly lower compared to the basic diet (*p* < 0.01). The remaining differences were not statistically significant.

The relative bioavailability of Cr from the studied dietary supplements under the influence of various types of diets varied within a very wide range of 0.79–8.08% (mean values). Thus, in the case of dietary supplement No. 1, a statistically significantly higher relative bioavailability of Cr from the standard diet compared to the basic (*p* < 0.001) and to a high-residue diet (*p* < 0.001) was demonstrated. Differences in the models using the basic and high-residue diets were insignificant. The average results of the relative bioavailability of Cr from product No. 1 ranged from 5.61 to 8.08%. As regards dietary supplement No. 2, the highest results were obtained for the basic diet, which were statistically significantly higher in comparison to the high-residue diet (*p* < 0.05). In models in which standard and high-residue diets were used, no significant differences were observed. The mean values of Cr bioavailability obtained for this dietary supplement were within the range of 5.97–6.44%. The average results for product No. 3, in which the element was present in the form of organic chromium yeast, fell within the range of 2.14–2.83%. The highest result was obtained in the model in which a high-residue diet was used, and this value was significantly higher in comparison to a standard diet (*p* < 0.001). Results obtained for dietary supplement No. 4 were the lowest among all studied products, ranging only from 0.79 to 0.96%. For this product, a statistically significantly lower bioavailability of Cr in the presence of the high-residue diet was demonstrated in comparison to the standard diet (*p* < 0.01) and to the basic diet (*p* < 0.01). The differences between the standard and the basic diet were insignificant. The average results for product No. 5 ranged between 3.72 and 6.45%. Results for the standard diet were significantly lower compared to the basic (*p* < 0.001) and high-residue diets (*p* < 0.001). The other differences were insignificant. In the case of dietary supplement No. 6, in which Cr was present in the form of chromium chloride, the average results were within the range of 1.02–1.78%. Significantly higher bioavailability of Cr was revealed in the model using the basic diet compared to the high-residue (*p* < 0.001) and standard diets (*p* < 0.001). Results between high-residue and standard diets were also statistically significant(*p* < 0.001).

### 3.2. Influence of Diet and Chemical Form on the Bioavailability of Cr

During the experiments, Cr used in the form of dietary supplements was present in three various chemical forms: chromium picolinate, chromium chloride, and organic chromium yeast. Each of these chemical forms was separately analyzed in terms of its bioavailability under the influence of the three types of diets used in the present study. The obtained results are presented in Table 4. Most dietary supplements with Cr present on the pharmaceutical market contain chromium picolinate, which is the most widely used organic form of this element. This compound is characterized by the high bioavailability of Cr, which was confirmed in the present study. The results obtained for chromium picolinate were by far the highest of all chemical forms subject to evaluation in this study. In the case of this chemical form of the element, the mean results were in the range of 5.96–6.15%. There were no significant differences between the results obtained for various diets, which means that a given type of diet has no influence on the bioavailability of Cr taken in the form of chromium picolinate. Chromium (III) chloride is the most popular form of inorganic chromium. This compound is relatively cheap and is thus widely used in the production of dietary supplements. The obtained results indicated very low Cr bioavailability from dietary supplements containing the compound in the form of chloride, as it was only 0.99–1.37%. Moreover, the results of the study showed a significant influence of the type of diet on Cr bioavailability—the highest results were obtained in models with a basic diet, which were significantly higher in comparison to standard (*p* < 0.01) and high-residue diets (*p* < 0.05). The differences between the results for standard and high-residue diets were insignificant. The values recorded for organic chromium yeast can be characterized as moderate regarding their bioavailability—higher in comparison to inorganic chromium (III) chloride and much lower in relation to chromium picolinate. The average results ranged between 2.14 and 2.83%. A significant influence of the type of diet was also shown—the highest results were obtained in models in which a high-residue diet was used. These results were significantly higher in comparison to the standard diet (*p* < 0.001). The differences between high-residue and basic diets were insignificant. The lowest results were obtained in models in which organic chromium yeast was mixed with the standard diet—these were significantly lower than in both basic (*p* < 0.001) and high-residue diets (*p* < 0.001).

### 3.3. Influence of the Pharmaceutical Form on the Bioavailability of Cr

Dietary supplements used in the present study were produced in three different pharmaceutical forms—lozenges, tablets, and coated tablets. The average bioavailability of Cr from each pharmaceutical form is presented in Table 5. In this table, data on the bioavailability of Cr from the diet were added only for statistical calculations. The obtained results revealed that the highest value of this parameter was achieved in the case of coated tablets (6.58%), and the lowest in the models using tablets (2.59%). It was also shown that a significantly higher relative bioavailability of Cr can be observed in experiments using coated tablets compared to tablets (*p* < 0.001) and compared to a natural diet (*p* < 0.001). The results obtained for lozenges were also significantly higher in comparison to models using tablets (*p* < 0.001) and compared to the bioavailability of Cr only from diet (*p* < 0.001). The results obtained for coated tablets and lozenges were insignificant.

## 4. Discussion

Cr is a biologically active trace element that has been perceived as necessary for the proper functioning of the human body for decades due to the multitude of metabolic processes in which it participates, especially by influencing the metabolism of proteins, fats, and carbohydrates. It has been described that Cr plays an important role in glucose metabolism, is involved in ensuring its appropriate level in the blood, is a component of many enzymes and a catalyst for numerous chemical reactions, and also takes part in antioxidant processes and in the functioning of the immune system [38]. However, some researchers have recently questioned its importance as an essential nutrient, as the status of Cr was not confirmed by the results of studies conducted on animals [39]. Therefore, it has been postulated that there is no clear evidence to consider Cr as an essential ingredient in a diet or as an essential element. It was also suggested that recommended intake standards should not be established for it [18].

Cr and its compounds are quite common in the environment. Products that contain a significant amount of this element include beef meat, dark chocolate, cheese, bread, vegetables (leek, green lettuce, radish, broccoli, and green beans), and fruits (kiwi, dried plums, apricots, apples, and oranges) [21].

Cr has a positive effect on human health as long as it is administered in the right dose. Its deficiency is inadvisable, while its excess may cause unfavorable symptoms [40]. It is claimed that Cr deficiency in the diet is unlikely to occur; however, mainly based on experimental studies conducted on animals, it has been shown that the symptoms of chromium deficiency may include decreased insulin activity and consequently decreased glucose tolerance and increased serum cholesterol levels. Additionally, disorders in the metabolism of proteins, carbohydrates, and lipids, atherogenic lesions in coronary arteries leading to the development of coronary artery disease, excessive appetite (including craving for sweets) and thirst, being overweight, problems with weight control, and even anxiety and depression have been observed [41,42,43]. However, there are few population studies that would indicate the existence of a relationship between insufficient Cr concentration in tissues or low intake and the occurrence of pathological conditions or diseases [44]. A review of the literature indicates that despite the promising results of animal studies on the impact of dietary Cr supplementation on lipid and carbohydrate metabolism, the conclusions drawn from the studies conducted in humans are still ambiguous. Taking into account the lack of results from well-planned, placebo-controlled, randomized studies conducted on large human populations, the use of dietary Cr supplementation to prevent overweight and obesity, reduce insulin resistance, and improve the blood lipid profile seems to be highly controversial [42]. This does not change the fact that dietary supplements containing Cr are still among the most popular ones on the market, for example in the USA, where sales exceeded USD 85 million in 2020 [45]. These are mainly used to reduce body weight and appetite for sweets. Research on the biological role of Cr continues to bring new results, and in many respects, this element is still considered essential for humans. Therefore, the need to expand knowledge of the bioavailability of this element and the impact of diet on this parameter seem to be of the utmost importance.

Interest in the content of Cr and its absorption from diet has been going on for decades. Several interesting conclusions were already reached in 1985, based on studies of the diets of 32 patients of both genders. It was found that for almost all diets (approximately 90%), the average daily intake of Cr was below the recommended intake (at that time, 50 µg), and the absorption of the element from the diet itself was inversely related to its content in the diet, which means that the more Cr is consumed, the less is absorbed [46]. A similar inverse relation between supply and the amount of absorption was observed by other researchers, who found that when the daily intake exceeded 40 µg, the absorption value reached a constant value of only 0.4%. It proves the great importance of the body’s ability to maintain homeostasis and endogenously regulate the level of absorption of individual substances, including essential nutrients [47]. In general, Cr is an element characterized by very low bioavailability. It is believed that there is no clear evidence that chromium in the form of natural complexes (e.g., found in brewer’s yeast) is absorbed better than in the form of simple inorganic salts [48]. The mechanism of Cr absorption is not fully understood, but it is probably absorbed through non-saturable passive diffusion [46]. Interestingly, it has been reported that this element is absorbed more strongly in women compared to men and that the degree of its retention in the body decreases significantly with age because elevated levels in the urine are observed in older people [49]. Elevated urinary Cr levels have also been observed to occur during various forms of stress, such as strenuous exercise, physical trauma, and infections [50].

The bioavailability of Cr also depends on the presence of substances that facilitate and hinder the absorption of this element. The former includes some amino acids, vitamin C, and nicotinic acid. In turn, substances that hinder absorption include ions of other metals that use the same carrier proteins in the absorption process and, as a result, compete for binding sites with chromium [51]. The elimination of absorbed Cr takes place mainly in urine in the form of chromodulin or other proteins that bind this element. Small amounts may also be excreted in sweat or bile [52,53]. According to Hambidge, the kidneys are considered the main organ involved in the physiological regulation of Cr homeostasis [54]. A significant amount of the element that is not absorbed in the intestine is excreted in the feces [19].

Recently, interest in Cr supplementation has increased significantly. However, it should be remembered that its effects on humans have never been proven, despite its popularity and wide usage [55]. Most supplements that are a mix of vitamins and minerals also contain Cr, most often at the level of 35–120 µg per dose. However, in the case of supplements containing only Cr, its amount may usually fall within the range of 200–500 µg [56]. In most cases, studies on the effect of increased Cr dosage were initially performed on animals, and after obtaining positive results, they were also repeated on patients (for example, those receiving total parenteral nutrition (TPN), patients with diagnosed diabetes) to alleviate disease symptoms [55]. However, as mentioned above, Cr supplements have not been found to produce significant results. Most of the clinical trials available are of short duration (six months or less) and do not control for confounders such as dietary intake and physical activity [23,45,57,58,59].

Cr is widely recognized as an element that has a positive effect on normalizing glucose levels and reducing body weight and is therefore proposed as a dietary component supporting the treatment of type 2 diabetes, overweight, and obesity. Under the influence of numerous pieces of literature, various products containing chromium salts (most often picolinate, nicotinate, and chloride) have been introduced to the dietary supplement market with a view to providing products that are supposed to be helpful in reducing body weight, suppressing the appetite for sweets, increasing the growth of muscle mass, and contributing to the normalization of serum glucose levels [18,19,60]. Clinical trial data also question the health-promoting properties of Cr compounds and their medical or dietary use. The American Diabetes Association (ADA) issued a statement in which it strongly calls for refraining from presenting Cr as an anti-diabetic substance, as there is no convincing evidence confirming such properties [61]. The FDA (Food and Drug Administration) is also critical of the impact of Cr picolinate supplementation on improving glucose tolerance and the possible use of its salts in the treatment of type 2 diabetes. The organization stated that, of the eight available health claims regarding the labeling of dietary supplements, only one small-scale study suggested that chromium picolinate might reduce the risk of type 2 diabetes [62]. The mentioned study was a placebo-controlled, double-blind study examining the effect of supplementation with 1 mg Cr/day (in the form of picolinate) on 29 obese people with a family history of diabetes. There was no effect of supplementation on body weight, fat tissue distribution, or its composition. However, a statistically significant increase in insulin sensitivity was observed after the 4th and 8th months of therapy [63].

Current studies suggest that the influence of Cr on the human body is a much more complicated issue than was previously thought. Recently, Zhao et al. revealed that co-supplementation of Cr and Mg improved glycemia and lipid levels. Moreover, the inflammatory response and oxidative stress profiles in patients were reduced [64]. Therefore, it is very important to evaluate not only the dietary intake of Cr but also other nutrients that may have a significant impact on the bioavailability of this element. This will allow a better understanding of the mutual proportions and impact of individual dietary components on their absorption in the gastrointestinal tract, which will contribute to their better use by the body.

As already mentioned above, the bioavailability of Cr is generally very low, although significant differences between individual studies should be taken into account, resulting from different methodologies for assessing this parameter. Studies on the bioavailability of Cr from preparations containing various chemical forms of this element have shown that organic Cr compounds (including chromium picolinate) are better absorbable than inorganic compounds (e.g., chromium (III) chloride) and are within a range of 10–20% [65]. Also, other studies suggested that Cr in the form of picolinate is the most preferable to use as a supplement because of its high bioavailability in comparison to other Cr salts [23,57]. The results of the present study have confirmed the highest bioavailability of Cr in the form of picolinate. However, the obtained values were below 10%, which is rather normal considering different experimental models. What is important is that our in vitro model confirmed the results of Król et al. experiments on animals [65], which may prove that in vitro models can be a cheap and fast method of initial evaluation of the bioavailability of various food components. The obtained results revealed that Cr bioavailability may be influenced not only by the chemical form and type of diet consumed but also by the quality of a given dietary supplement, its formulation, and, above all, the type of excipients influencing the release of individual ingredients. It was shown that significant differences can be found not only between various types of diets but also between particular products that contain the same chemical form of Cr. Given the average results (Table 4), the highest bioavailability of Cr was determined in the presence of a high-residue diet, but the differences were insignificant in comparison to both standard and basic diets. Therefore, it can be concluded that the bioavailability of Cr was the highest in the case of picolinate, but the type of diet had no influence on the value of this parameter.

As regards the inorganic form, the results of the present study showed an average bioavailability of Cr ranging between 0.99 and 1.37%. The significant influence of the type of diet was proven, and the highest results were observed in models using the basic diet, followed by high-residue and standard diets. It is hard to find a similar study in the literature on the subject to compare the obtained results with those of other authors, but our previous research, performed for Se, also indicated a positive influence of a basic diet on the bioavailability of this element [30]. In general, inorganic forms of Cr are characterized by the lowest values of bioavailability. Król et al. [65] showed the relative bioavailability of inorganic forms of Cr at the level of 0.4–3%, which was very low in comparison to picolinate. On the other hand, studies conducted by Laschinsky et al. on rats showed that inorganic Cr compounds, including chromium (III) chloride, may provide higher relative bioavailability values for this element compared to organic forms. This is because the fraction of absorbed but directly excreted Cr cannot perform a metabolic function in the body, and therefore, the true absorption of the element, understood as the amount of Cr retained in the body added to the amount excreted in urine and feces, may result in an overestimation of its bioavailability. Consequently, compounds such as chromium (III) chloride provide the same or even higher amounts of Cr than chromium picolinate or chromium nicotinate, but their retention in the body is lower in comparison to organic forms [66]. However, the above thesis is in contradiction to the literature because it was previously proven that high urinary Cr excretion was balanced by an increase in the amount of this element retained in the body [67]. The best bioavailability of Cr from chromium picolinate was stated according to studies conducted on volunteers supplemented with this compound, among whom particularly high urinary excretion of chromium was observed, which proved high absorption of this element [68]. Moreover, according to model calculations, it was directly predicted that trivalent Cr taken orally in the form of picolinate might accumulate in human tissues, which proves its high retention in the human body [69]. The highest rate of Cr excretion in urine in the case of supplementation with chromium picolinate was also observed in studies on the absorption of this element from various chemical forms in dietary supplements available on the market among volunteers who were orally administered doses of 200 μg of Cr while examining its 24 h excretion with urine [70]. This further strengthens the view that chromium picolinate has always resulted in the highest urinary excretion of this element, leading to the conclusion that this Cr salt is the best-absorbed chemical form [66], which was proven in the current study, although using a completely different experimental model.

In the current study, we have also evaluated the bioavailability of Cr from organic chromium yeast. This form was considered moderately effective, as the bioavailability of Cr from this form was much higher in comparison to chromium (III) chloride but much lower in relation to picolinate. The type of diet also had a significant influence. The highest bioavailability was determined in the case of a high-residue diet and the lowest for the standard diet. According to the manufacturer’s declarations, this form of Cr should have a bioavailability that is 10 times higher than that of other sources of the element. The obtained results did not confirm this thesis because the determined bioavailability of Cr from the diet was at a similar level, and chromium picolinate was shown to be the best source of this element for any type of diet consumed.

Dietary supplements in the form of coated tablets were shown to be the best sources of Cr in comparison to lozenges and tablets. However, considering the small number of products used in the study, it is an initial observation that needs further research. It is also hard to compare these data with other studies because such research has not yet been performed.

Over the years, the opinion on how important Cr is in the human diet and what role it plays in maintaining the proper functioning of the body has been constantly changing [71]. However, this does not change the fact that it is important to know certain relationships between the content of this element in food and its actual bioavailability. The most reliable estimate of the bioavailability of substances and elements supplied through food and in the form of supplements containing preferred substances is possible thanks to the most accurate mapping of digestive processes taking place in a living organism. The in vitro procedure conducted outside the body is not only faster and much cheaper but also less problematic because it does not require animals or humans. This method is also free from ethical concerns and health risks when analyzing toxic elements. This study also has some limitations. Firstly, considering the use of three types of diet and the resulting large number of experimental models, we used only six dietary supplements in the study, representing the chemical forms that are most commonly used on the market. Secondly, supplements are administered in different ways. Often on an empty stomach, at different times, and with different meals. Therefore, a perfect reproduction of such behaviors using the in vitro model and based on analytical determinations is basically impossible. In designing the types of diet used in the study, we tried to illustrate the possible impact of diet composition on the bioavailability of the element from supplements; therefore, three types of major diets used in the nutrition of healthy people were designed and reconstructed. Moreover, a lack of representativeness regarding pharmaceutical forms of particular products reduces the reliability of the obtained results. The unquestionable strengths of the research carried out include the fact that it was a typical analytical study using modern research techniques. The obtained results were supported by a thorough statistical analysis, which enabled the authors to draw reliable conclusions. An additional strength of the research is that we used products from several series in order to obtain more representative results.

## 5. Conclusions

The presented study showed the possibility of using a simulated in vitro digestion model to evaluate the bioavailability of Cr from dietary supplements and the impact of the type of diet on the value of this parameter, which was the major aim of the conducted research. It was shown that Cr picolinate was characterized by the highest bioavailability, while the type of diet had no influence on that value. A high-residue diet characterized by a high content of fiber positively influenced the bioavailability of Cr from dietary supplements containing organic forms of the element. It was proven that the bioavailability of Cr in the inorganic form (chromium chloride) was much lower in comparison to organic forms and natural diets. A basic diet was shown to positively affect the bioavailability of Cr in the form of chloride. This diet was characterized by a moderate content of basic nutrients and dietary fiber, and therefore, it can be assumed that a well-balanced diet containing foods from all major food groups may have a positive impact on the bioavailability of chromium (III) chloride, but this issue needs further research. The innovation of the conducted research is the demonstration of a significant impact of the food matrix on the value of Cr bioavailability in conditions of simulated in vitro digestion because most studies of this type assess only the value of this parameter, where in most cases, the supplements are not taken on an empty stomach but enter the digestive tract filled with various amounts of food. Therefore, the results of the current study also have important practical meaning for consumers, as the research conducted may help optimize the use of Cr contained in dietary supplements by the human body. This study has shown that the use of simulated in vitro digestion using modern analytical techniques can be a cheap and quick tool for assessing the bioavailability of Cr from various products, including dietary supplements. The presented model can also be widely modified to study the influence of various parameters on the bioavailability value, such as the type of diet, which was the main goal of the conducted research.

## Figures and Tables

**Table 1:** Detailed characteristics of dietary supplements used in the study.

	Cr		
Product No	Chemical Form	Supplement Type	Pharmaceutical Form
1	chromium picolinate	vitamin–mineral	coated tablets
2	chromium picolinate	vitamin–mineral	lozenges
3	organic chromium yeast	single-mineral	tablets
4	chromium chloride ^1^	vitamin–mineral	tablets
5	chromium picolinate	single-mineral	tablets
6	chromium chloride ^1^	vitamin–mineral ^2^	tablets

^1^ CrCl_3_; ^2^ enriched with plant extracts

**Table 2:** Selected parameters of the applied analytical determinations.

Parameter	Cr (GF-AAS)	Cr (ICP-OES)
Reference value (mg/kg)	0.15	0.15
Determined value (mg/kg)	0.15	0.16
	0.14	0.17
	0.15	0.18
	0.17	0.14
	0.17	0.18
	0.16	0.17
**Average**	**0.16**	**0.17**
SD	0.012	0.015
RSD (%)	7.50	8.82
Recovery (%)	106.7	113.3
LOD (µg/kg)	0.57	1.20
LOQ (µg/kg)	2.20	4.30

SD—standard deviation; RSD—relative standard deviation; LOD—limit of detection; LOQ—limit of quantification.

**Table 3:** Influence of various types of diets on the bioavailability of Cr from the dietary supplements used in the study.

Dietary Supplement No	Chemical Form	Diet	M	Me	Min	Max	IQR	SD	One-Way ANOVA		Tukey’s Post Hoc Test Results		
									F	*p*	Group 1	Group 2	*p*
Without (%)	---	Standard	2.97	2.91	2.46	3.55	0.40	0.36	6.06	<0.01	Standard	Basic	<0.01
		Basic	3.70	3.81	3.02	4.77	0.88	0.59			Standard	High residue	>0.05
		High residue	3.22	3.16	2.73	3.80	0.19	0.36			Basic	High residue	>0.05
1	chromium picolinate	Standard	8.08	8.03	6.58	9.98	0.79	0.96	42.06	<0.001	Standard	Basic	<0.001
		Basic	5.61	5.69	5.12	6.09	0.51	0.35			Standard	High residue	<0.001
		High residue	6.04	5.95	5.74	6.43	0.42	0.27			Basic	High residue	>0.05
2	chromium picolinate	Standard	6.08	6.18	5.54	6.29	0.27	0.27	5.44	<0.05	Standard	Basic	>0.05
		Basic	6.44	6.29	5.99	7.05	0.75	0.41			Standard	High residue	>0.05
		High residue	5.97	5.97	5.65	6.43	0.21	0.24			Basic	High residue	<0.05
3	organic chromium yeast	Standard	2.14	2.09	1.58	2.69	0.43	0.33	26.73	<0.001	Standard	Basic	<0.001
		Basic	2.78	2.79	2.59	2.91	0.20	0.12			Standard	High residue	<0.001
		High residue	2.83	2.78	2.64	3.09	0.26	0.16			Basic	High residue	>0.05
4	chromium (III) chloride	Standard	0.96	0.95	0.91	1.03	0.05	0.05	7.68	<0.01	Standard	Basic	>0.05
		Basic	0.96	0.95	0.74	1.18	0.31	0.17			Standard	High residue	<0.01
		High residue	0.79	0.79	0.74	0.84	0.08	0.04			Basic	High residue	<0.01
5	chromium picolinate	Standard	3.72	3.71	3.27	4.18	0.39	0.29	189.03	<0.001	Standard	Basic	<0.001
		Basic	6.31	6.41	5.81	7.05	0.50	0.40			Standard	High residue	<0.001
		High residue	6.45	6.45	6.10	6.85	0.55	0.30			Basic	High residue	>0.05
6	chromium (III) chloride	Standard	1.02	1.02	0.96	1.08	0.05	0.04	146.51	<0.001	Standard	Basic	<0.001
		Basic	1.78	1.73	1.65	1.95	0.18	0.11			Standard	High residue	<0.001
		High residue	1.37	1.37	1.20	1.53	0.16	0.12			Basic	High residue	<0.001

**Table 4:** Bioavailability of Cr, taking into account the chemical form, under the influence of various types of diets.

Chemical Form	Diet	M	Me	Min	Max	IQR	SD	One-Way ANOVA		Tukey’s Post Hoc Test Results		
								F	*p*	Group 1	Group 2	*p*
chromium picolinate(%)	Standard	5.96	6.18	3.27	9.98	3.83	1.91	0.22	>0.05	Standard	Basic	>0.05
	Basic	6.12	6.09	5.12	7.05	0.70	0.53			Standard	High residue	>0.05
	High residue	6.15	6.11	5.65	6.85	0.52	0.34			Basic	High residue	>0.05
chromium (III) chloride (%)	Standard	0.99	0.99	0.91	1.08	0.08	0.05	7.17	<0.01	Standard	Basic	<0.01
	Basic	1.37	1.42	0.74	1.95	0.78	0.45			Standard	High residue	>0.05
	High residue	1.08	1.02	0.74	1.53	0.58	0.31			Basic	High residue	<0.05
organic chromium yeast (%)	Standard	2.14	2.09	1.58	2.69	0.43	0.33	26.73	<0.001	Standard	Basic	<0.001
	Basic	2.78	2.79	2.59	2.91	0.20	0.12			Standard	High residue	<0.001
	High residue	2.83	2.78	2.64	3.09	0.26	0.16			Basic	High residue	>0.05

**Table 5:** Bioavailability of Cr, taking into account the pharmaceutical form of the dietary supplements.

Pharmaceutical Form	M	Me	Min	Max	IQR	SD	Kruskal–Wallis ANOVA	
							H	*p*
Diet (%)	3.29	3.15	2.46	4.77	0.84	0.53	84.82	<0.001
Coated tablets (%)	6.58	6.09	5.12	9.98	2.00	1.25		
Losenges (%)	6.16	6.13	5.54	7.05	0.32	0.37		
Tablets (%)	2.59	1.91	0.74	7.05	2.16	1.92		

M—arithmetic mean; Me—median; Min—minimum; Max—maximum; IQR—quartile range; SD—standard deviation; H—test statistic value for the ANOVA test.

## Data Availability

Data are contained within the article or supplementary material.

## Supplementary Materials

The following supporting information can be downloaded at: https://www.mdpi.com/article/10.3390/nu16071022/s1. Table S1: Composition of diets used in the study; Table S2. Selected nutritional parameters of diets used in the study; Table S3. Operating parameters in the ICP-OES method; and Table S4. Operating parameters in the GF-AAS method.
