# Supplementary material for: In Vitro Evaluation of Bioavailability of Cr from Daily Food Rations and Dietary Supplements from the Polish Market
Source: Nutrients. 2024 Mar 31;16(7):1022. doi: 10.3390/nu16071022 (PMC11013223; doi:10.3390/nu16071022)
Supplement: Supplementary file 1 [file nutrients-16-01022-s001.zip › nutrients-2924467-supplementary.pdf]

SUPPLEMENTARY FILE

In vitro evaluation of bioavailability of Cr from daily food rations and dietary supplements

Piotr Bawiec[1], Jan Sawicki[2], Paulina Łasińska-Pracuta[1], Marcin Czop[3], Ireneusz Sowa[2] , Paweł Helon[4], Karolina Pietrzak[1] and Wojciech Koch[1,*]

[1] Department of Food and Nutrition, Medical University of Lublin, 4a Chodźki Str., 20-093 Lublin, Poland; piotr.bawiec@wp.pl (P.B.); paulia_lasinska@interia.pl (P.Ł.-P.); karolinapietrzak94@gmail.com (K.P.)
[2] Department of Analytical Chemistry, Medical University of Lublin, 4a Chodźki Str., 20-093 Lublin, Poland; jan.sawicki@umlub.pl (J.S.); ireneusz.sowa@umlub.pl(I.S.)
[3] Department of Clinical Genetics, Medical University of Lublin, Radziwiłłowska 11 Str., 20-080 Lublin, Poland; marcin.czop@umlub.pl (M.C.)
[4] Branch in Sandomierz, Jan Kochanowski University of Kielce, Schinzla 13a Str., 27-600, Sandomierz, Poland; phelon@ujk.edu.pl (P.H.)

* Correspondence: kochw@interia.pl; Tel.: +48-81-448-7142

Table S1. Composition of diets used in the study [30].

Type of the meal	Food product/meal	Amount (g/mL)
	**Basic diet**	
Breakfast	cereal coffee with milk	250
	white bread	80
	cottage cheese	110
	Onion	10
	cucumber	30
	butter	10 g
Second breakfast	mixed bread (wheat-rye flour)	60
	chicken pate	130

	apple	150
	black tea infusion	250
Lunch	vegetable soup (including carrots, celery, parsley, cauliflower)	400 (vegetables – 100 g)
	poultry chop	150
	potatoes	300
	red cabbage salad	150
	grated strawberry compote	250 (strawberries -30 g)
Dinner	meatballs in sauce	120
	pasta	120
	salad (carrot, apple, mayonnaise)	100
	yeast cake with crumble	50
	black tea infusion with milk	250
**Standard diet**		
Breakfast	ham sausages	150
	mixed bread (wheat-rye flour)	90
	cocoa with milk	250
	mustard	20

Second breakfast	gouda cheese	60
	crispbread	30
	coffee infusion with milk	150
Lunch	tomato soup with pasta	400
	Potatoes	300
	grilled cod	200
	salad with sauerkraut	150
	compote	250
	coffee infusion with milk	150
	milk chocolate with nuts	30
Dinner	mixed bread (wheat-rye flour)	80
	sausages	110
	pickled cucumber	80
	black tea infusion	250
**High-residue diet**		
Breakfast	oatmeal in milk	oat flakes – 50 g<br>milk – 350 g
	wholemeal bread	80

	cottage cheese	80
	jam	20
Second breakfast	mixed bread (wheat-rye flour)	80
	rennet cheese	40
	ham	440
	tomatoes	150
	banana	100
	black tea infusion	250
Lunch	beetroot soup	400 (beetroot – 30 g; vegetables: carrots, parsley, celery – 25 g)
	pork chop	150
	potatoes	300
	boiled vegetables (carrots with peas 1+1 with breadcrumbs and butter)	300
	apple	150
	strawberry compote	200
Dinner	mixed bread (wheat-rye flour)	80
	chicken ham	80

	red pepper	150
	butter	10 g
	coffee infusion with milk	150

Table S2. Selected nutritional parameters of diets used in the study [30].

Parameter	Diet		
	**Basic**	**Standard**	**High-residue**
Proteins (g)	116.8 (17.3% of E*)	153.9 (22.2% of E)	143.5 (18.5% of E)
Fats (g)	109.2 (36.4% of E)	129 (41.9% of E)	117.1 (34% of E)
Carbohydrates (g)	338 (46.3% of E)	272.2 (35.8% of E)	416 (47.5% of E)
Fiber (g)	28.6	24.6	50.2
Vitamin A (μg)	2960	500	4600
Vitamin C (μg)	80.6	54.8	304.7
Vitamin E (mg)	17.2	15.9	24.7
Calcium (mg)	568.7	1203	1465
Sodium (mg)	2370	4865	3290
Potassium (mg)	4877	5615	6837
Magnesium (mg)	361	562.9	693
Iron (mg)	14.3	17.2	21.7
Energy (kcal)	2699	2770	3099
Total weight (g)	2970	2750	3285

* Percentage of energy

Table S3. Operating parameters in the ICP-OES method.

Analytical line, reading time	Se 267.716 nm, 3 s
Signal reading type	axial
Signal integration	3 pix
Plasma generator power	1300 W
Coolant gas flow rate	14 $L{\cdot}min^{-1}$
Auxiliary gas flow rate	0.5 $L{\cdot}min^{-1}$
Carrier gas flow rate	0.6 $L{\cdot}min^{-1}$
Sample flow rate	1.0 $L{\cdot}min^{-1}$

Table S4. Operating parameters in the GF-AAS method.

Analytical line, reading time	Cr 357.869 nm, 5 s
Time and temperature program for graphite furnace operation	
drying	80°C, 20 s
drying	90°C, 20 s
drying	110°C, 10 s
pyrolysis	350°C, 20 s
pyrolysis	1300°C, 10 s
atomization	2400°C, 5 s
cleaning	2600°C, 4 s
Matrix modifier	0.05% Mg, 5 μL
Signal integration	3 pix
Sample volume	20 μL
